# Canine Spontaneous Head and Neck Squamous Cell Carcinomas Represent Their Human Counterparts at the Molecular Level

**DOI:** 10.1371/journal.pgen.1005277

**Published:** 2015-06-01

**Authors:** Deli Liu, Huan Xiong, Angela E. Ellis, Nicole C. Northrup, Kevin K. Dobbin, Dong M. Shin, Shaying Zhao

**Affiliations:** 1 Department of Biochemistry and Molecular Biology, Institute of Bioinformatics, University of Georgia, Athens, Georgia, United States of America; 2 College of Veterinary Medicine, University of Georgia, Athens, Georgia, United States of America; 3 Department of Biostatistics, University of Georgia, Athens, Georgia, United States of America; 4 Winship Cancer Center, Emory University School of Medicine, Atlanta, Georgia, United States of America; Dana Farber Cancer Institute, UNITED STATES

## Abstract

Spontaneous canine head and neck squamous cell carcinoma (HNSCC) represents an excellent model of human HNSCC but is greatly understudied. To better understand and utilize this valuable resource, we performed a pilot study that represents its first genome-wide characterization by investigating 12 canine HNSCC cases, of which 9 are oral, via high density array comparative genomic hybridization and RNA-seq. The analyses reveal that these canine cancers recapitulate many molecular features of human HNSCC. These include analogous genomic copy number abnormality landscapes and sequence mutation patterns, recurrent alteration of known HNSCC genes and pathways (e.g., cell cycle, PI3K/AKT signaling), and comparably extensive heterogeneity. Amplification or overexpression of protein kinase genes, matrix metalloproteinase genes, and epithelial–mesenchymal transition genes *TWIST1* and *SNAI1* are also prominent in these canine tumors. This pilot study, along with a rapidly growing body of literature on canine cancer, reemphasizes the potential value of spontaneous canine cancers in HNSCC basic and translational research.

## Author Summary

Head and neck squamous cell carcinoma (HNSCC) represents the sixth leading cancer by incidence in humans; thus, developing effective therapeutic interventions is important. Although great advance has been made in our understanding of the biology of HNSCC over the past several decades, translating the research findings into clinical success has been frustratingly slow, and anticancer drug development remains a lengthy and expensive process. A significant challenge is that drug effects in current preclinical cancer models often do not predict clinical results, and there lacks translational models that can bridge the gap between preclinical research and human clinical trials. Here we report a pilot study that represents the first genome-wide characterization of spontaneously occurring HNSCCs in pet dogs. The study reveals a strong dog-human molecular homology at various levels, indicating the likelihood that spontaneous canine HNSCC molecularly represents its human counterpart. If conclusions of this pilot study are validated with a large sample size and more efforts are put into building better resource and infrastructure for canine cancer research, spontaneous canine HNSCCs could effectively serve as a much-needed translational model that bridges the gap between preclinical research and human trials.

## Introduction

During the past several decades, great advances have been made in our understanding of the biology of head and neck squamous cell carcinoma (HNSCC) [1–8]. For example, owing to the rapid development in next-generation sequencing and other high throughput technologies, the cancer genome atlas (TCGA) [1] and others [2–4] have characterized hundreds of human HNSCC cases and discovered significantly altered genes and pathways (cell cycle, PI3K signaling, etc.). However, translating these research findings into clinical success has been frustratingly slow, and drug development remains a lengthy and expensive process [9], with costs currently estimated at over US$1 billion to bring a new drug to market [9]. With biomarker-based clinical trials not as advanced and half as many clinical trials currently available as lung or breast cancer [5], the issue is even more serious for HNSCC. One significant challenge is the lack of effective predictive models [9].

Current widely-used HNSCC models [10–12] include: 1) cell line and xenograft models; 2) genetically engineered mouse models; and 3) carcinogen-induced models. While these models have made spectacular contributions in our molecular understanding of HNSCC and are clearly indispensable, they have issues and generally do not fully represent the great complexity and heterogeneity of human HNSCC. First, cell lines and subcutaneous xenograft models lack the specific interactions between tumor cells and their native microenvironment that significantly influences carcinogenesis. Orthotopic xenograft models allow tumor development in places that are closer to the natural anatomic site [13], but the tumors still do not arise from the native head and neck epithelium, a significant issue considering the importance of cells of origin in cancer [14]. Second, genetically engineered mouse models often manipulate only one or a few major driver genes (e.g., *TP53*, *CCND1*, *KRAS*, *AKT*, etc.) [12], and normally fail to recapitulate the full alteration spectrum of human HNNCC that typically involves hundreds of genes per tumor [1–4]. Third, carcinogen-induced models such as the 7,12-dimethylbenz[a]anthracene (DMBA)–induced tumors in Syrian hamster cheek pouch do not grossly or histologically resemble human oral carcinomas [12]. Chronic administration of 4-nitroquinoline 1-oxide (4NQO), which can cause DNA adduct formation and Hras mutation, induces oral tumors in rodents [12]. However, these models do not represent the invasive pathology of human HNSCC [12]. In summary, the current widely-used HNSCC models usually fail to accurately recapitulate the full spectra of the biology, histopathology, complexity, and heterogeneity of HNSCC in humans. This, combined with the body size difference between the human and the rodents, make these models often incapable of accurately predicting the drug efficacy and toxicity in human patients [9]. Thus, HNSCC models that can bridge these models and human clinical trials are critically missing and urgently needed at present.

Spontaneously occurring HNSCCs in pet dogs may bridge this gap as they overcome many of the issues of the current HNSCC models discussed above [15–30]. First, unlike genetically-engineered or xenograft rodent models, these cancers are naturally occurring and heterogeneous, capturing the essence of human cancers. Second, as companion animals, dogs share the human environment and are exposed to many of the same carcinogens in a similar fashion, unlike carcinogen-induced cancers. Third, dogs better resemble humans in biology, e.g., with similar telomere and telomerase activities [31] and frequent occurrence of spontaneous epithelial cancers [15], than mice [32]. This further allows a closer resemblance in biology of HNSCC. Notably, the availability of a genome assembly nearly as accurate as the mouse or rat genome [33,34], in contrast to that available for another companion animal, the cat, make many analyses possible with canine cancers but not with feline cancers.

HNSCCs are relatively frequent in the dog and like their human counterparts, the oral cavity is a common site, and oral SCC is the 2^nd^ most common oral malignant tumor in dogs [15,35–37]. The prevalence rate for nontonsillar oral SCC is estimated at 6.4–7.3 per 100,000 [15], compared to the average incidence rate of human head and neck cancers at 8.8 and 5.1 per 100,000 men and women respectively [38]. For canine tonsillar SCC, the prevalence rate varies dramatically, ranging from 91–120 per 100,000 in large cities such as London and Philadelphia to nearly none in rural areas [15]. Critically, numerous anatomic and clinical similarities of HNSCC are noted between the human and the dog [15,35]. For example, as in humans, canine HNSCCs are invasive with metastasis occurring late in disease and tumor cells spreading to the regional lymph nodes and occasionally to the lung, and local disease recurrence is common in many patients. An interesting exception to these resemblances is that canine tonsillar SCCs are more aggressive and often metastatic (73%) compared to those in other locations, which however is observed in humans. Although far from being as extensively characterized as in human HNSCCs, papillomavirus DNA is also detected in canine oral SCCs [39]. Lastly, similar treatment schemes are practiced in the two species, which include surgery, radiation therapy, and chemotherapy with agents such as piroxicam and carboplatin [40,41].

Because of the features described above, along with a large population of pet dogs (~70 million estimated in the US alone), canine HNSCC could potentially be a useful and practical model that significantly speeds up bench to bedside translation. However, a major obstacle for this realization is that canine HNSCC is markedly understudied. Our current literature search indicates that not a single canine HNSCC genome or transcriptome has ever been investigated by sequencing, microarray, or other strategies. This drastically differs from other canine cancers such as lymphoma [42,43], leukemia, and osteosarcoma [26], of which hundreds of cancer genomes have been characterized via array comparative genomic hybridization (aCGH) or molecular cytogenetic analyses. Thus, unlike the better-studied canine cancers, there are simply insufficient data to evaluate the extent of molecular similarity between canine HNSCC and its human counterpart, a key factor in determining the usefulness of canine HNSCC in basic and translational research. To address this obstacle, we set out to conduct a pilot study that represents the first genome-wide characterization of spontaneous canine HNSCC.

## Results

### Twelve spontaneous canine HNSCC, including nine oral SCC, cases were investigated

**Fig 1:**
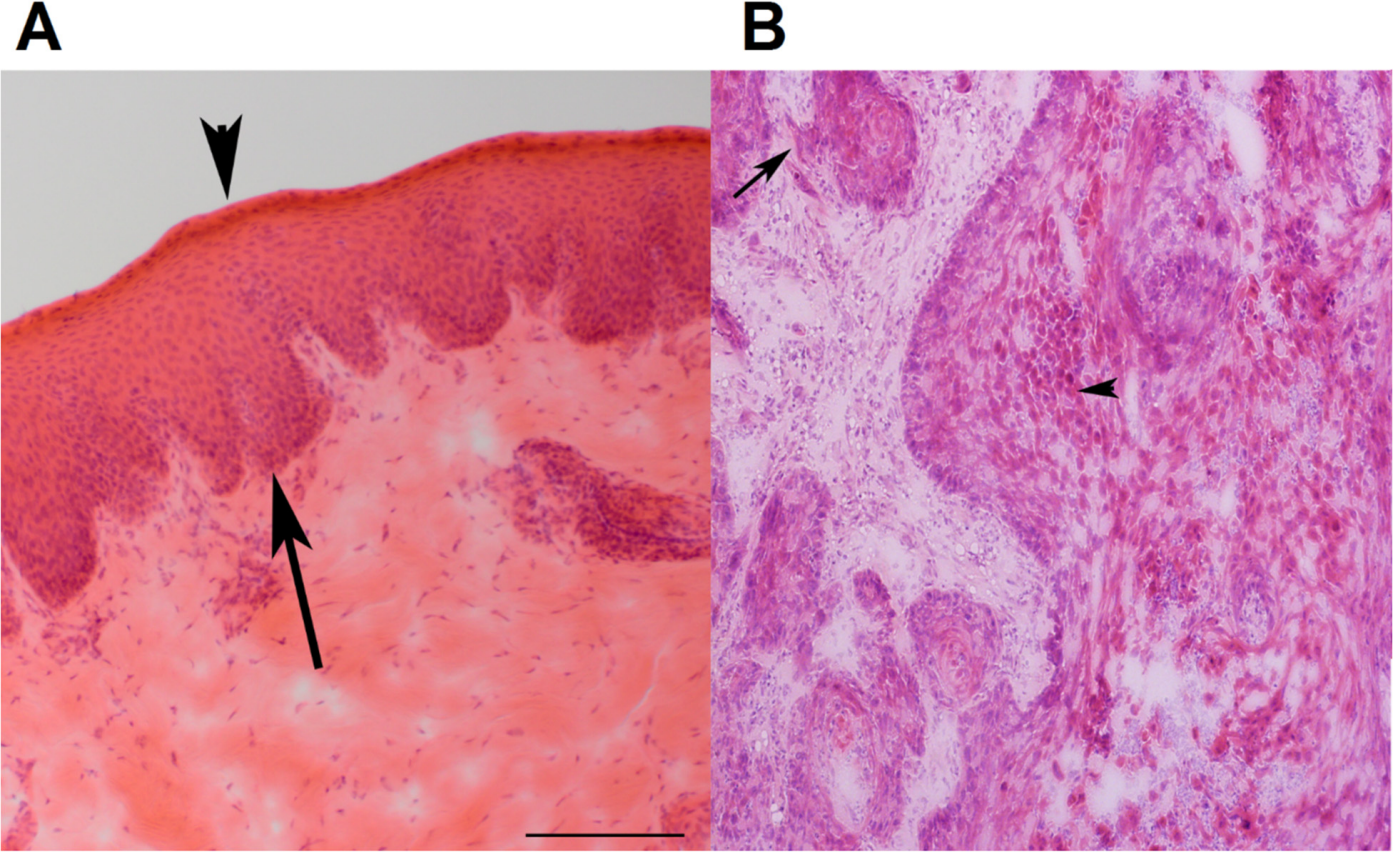
Representative H&E stained images of canine normal squamous epithelium and SCC of the oral cavity. (A) Normal squamous epithelium of the oral mucosa (from case 240). Epithelium is between the arrow and the arrowhead. The arrow indicates the basal layer, while the arrowhead designates the stratum corneum. (B) SCC of the oral mucosa (from case 240). The arrow points to an area with loss of a distinct basal layer and basement membrane. The arrowhead indicates keratinizing squamous cells. The images are at 100X magnification.

We characterized 12 spontaneous canine HNSCC cases by genome-wide analyses including high density aCGH and RNA-seq. These cancers come from different dog breeds, including four Labrador retrievers, three mixed breeds, and others listed in S1A Table. Among the 12 HNSCC cases, nine are oral (oSCCs) and all nontonsillar, with one from the buccal mucosa, three from the tongue, and five from the gingiva. The remaining three cases are from the nasal planum, the nostril, and the eye (the ocular adnexa). The tumors are all invasive (Fig 1A and 1B), and most are well-differentiated although some are more disorganized than others (S1A Table).

Furthermore, two cases appear to be papillomavirus-positive, based on the analysis described in later sections.

### aCGH analysis reveals dog-human homology in genomic copy number abnormality

**Fig 2:**
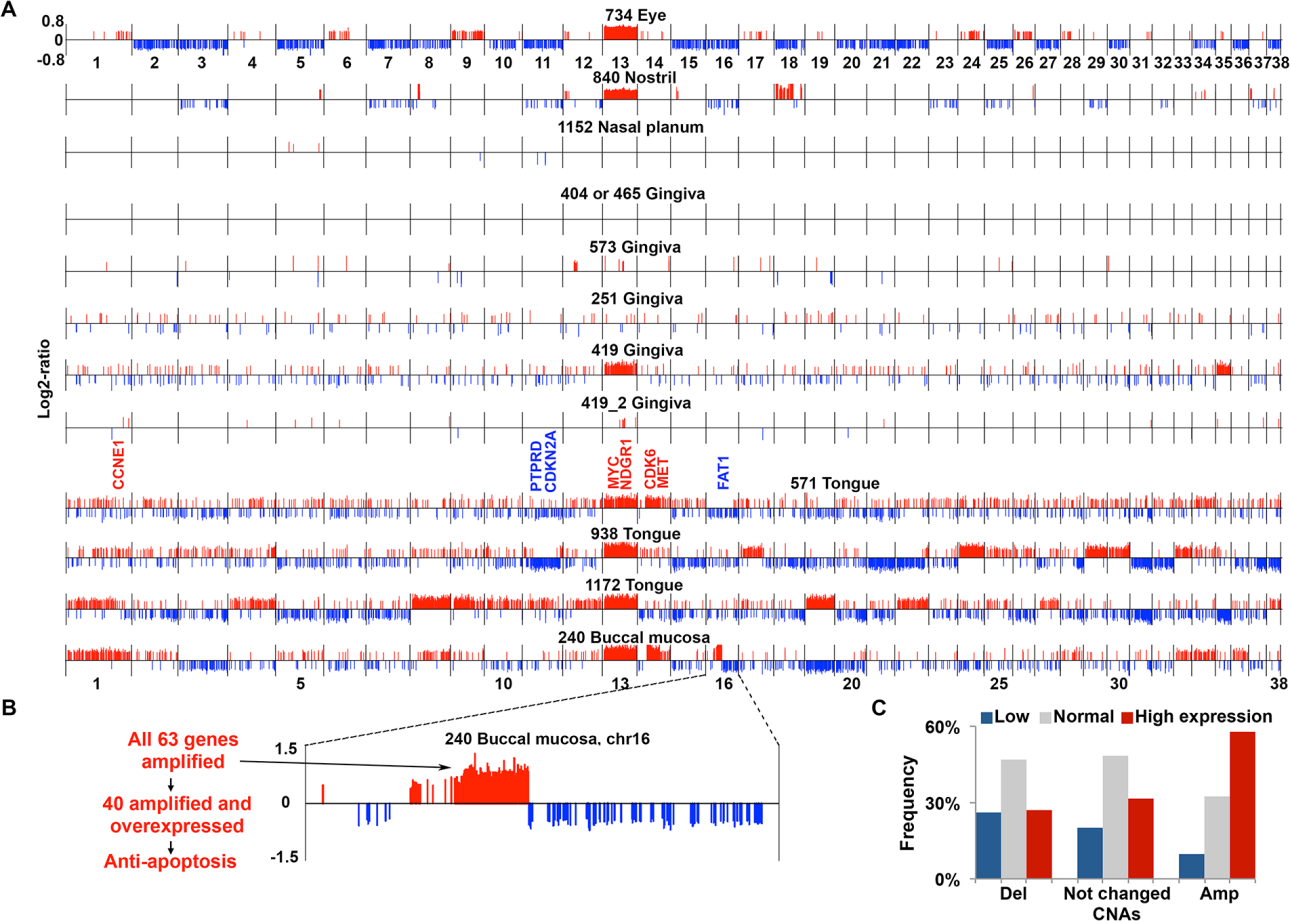
aCGH analysis reveals extensive CNAs in the majority of canine HNSCCs. (A) CNAs found in canine HNSCCs. Each tumor genome, with the tumor ID and location indicated on the top, is shown as a continuous line consisting of 38 canine autosomes separated by vertical black lines, with the chromosome numbers indicated. Within each chromosome, red/blue vertical lines above/below the chromosome indicate amplifications (red) or deletions (blue) respectively, with the line height representing the log2-ratio of the amplified or deleted segment. Tumors 419 and 419_2 are from the same case (ID 419), with 419_2 being less advanced. Notable amplified (red) or deleted (blue) genes, e.g., *MYC*, etc. are indicated.(B) Chromosome 16 of tumor 240 is enlarged to show a focal amplification, located at 20.8–30.7 Mb of chromosome 16 and harboring 63 genes as indicated, to exemplify that many of the amplified genes are also overexpressed and associated with tumor-promoting function. (C) Integration of genes’ copy number status and mRNA expression level. Genes (11,821 total) with FPKM (fragments per kilobase of exon per million mapped fragments) of ≥ 1 in at least one of the tumors subjected to RNA-seq were sorted into three groups based on the analysis indicated in (A): deleted (Del), unchanged, or amplified (Amp) as shown on the X-axis. Then, each group of genes was further divided into three subgroups based on the mRNA expression level: low, normal, and high determined as described [2], with the percentages shown in the Y-axis. See also S2B Table.

Analogous to human HNSCCs [1,2,6], aCGH analysis reveals variations in the extent of genomic copy number abnormality (CNA) among these canine tumors. Specifically, while seven canine HNSCCs harbor extensive CNAs, the remaining five tumors have hardly any CNAs in their genomes (Fig 2A and S1B Table). In some cases, the variation in the CNA prevalence is clearly related to the cancer progression stage (see tumors 419 and 419_2 in Fig 2A). In other cases however, additional factors may also have contributed. For example, tumor 1152 lacks CNAs but is at a tumor-progression stage similar to those with extensive CNAs (Fig 2A). Interestingly, among the nine oSCCs, those with a buccal mucosa or tongue location harbor significantly more CNAs than those located in the gingiva (Fig 2A and S1A and S1B Table).

As in human HNSCCs [1,2,6], both focal and broad CNAs were detected in the canine tumors (Figs 2A and S1). For example, tumor 240 harbors a focal amplification of ~10Mb located on chromosome 16, increasing the copy number of nearly all 63 genes inside by 2-fold and more (Fig 2B and S1C Table). Of these genes, 40 are also overexpressed (see later sections), among which “negative regulation of apoptosis” and “endopeptidase activity” are the most enriched functional groups. Indeed, anti-apoptosis associated genes *IKBKB*, *POLB*, *SFRP1* and *FNTA*, as well as endopeptidase genes *ADAM9* and *PLAT* encoded in the region are both amplified and overexpressed (S1C and S1D Table) (these genes are also recurrently amplified in human cancers including HNSCC according to cBioPortal [44] at www.cbioportal.org). Notably, 42 of the 63 genes are also significantly amplified in human HNSCC based on the TCGA study [1] (which will be examined further in a later section). These observations support that this focal amplification may have contributed to the pathogenesis of tumor 240. Meanwhile, broad events such as recurrent gain of canine chromosome 13, observed in seven tumors out of 12 total (Fig 2A), were seen as well (although other canine cancer types also harbor chromosome 13 gain [21,43]). The first 40Mb of canine chromosome 13 is syntenic to the last 48Mb of human chromosome 8q, which encodes genes including *MYC* and is one of the most recurrently amplified sites in human HNSCC [1,2] and other cancer types [45,46]. Consistent with published studies [21,43], these observations support common cancer drivers between the human and the dog.

In most canine HNSCCs, more amplifications than deletions were found, causing more genomic sequences and genes to be amplified than deleted (Fig 2A and S1B Table). Amplifications are, on average, also larger than deletions (S2 Fig and S1B Table). Importantly, a better correlation between the copy number status and the expression level (see later sections) was observed for amplified genes than deleted genes (Fig 2C), supporting that amplified regions harbor more cancer drivers than deleted regions. This is also consistent with human findings [2].

Many known human HNSCC genes [1,2,47–49] are also amplified/deleted in the canine tumors. Examples include recurrent amplification of known oncogene *MYC*; protein kinase genes *MET*, *PTK2* (also known as *FAK1*) and *FGFR1;* apoptosis-related *FADD;* histone lysine methyltransferase gene *WHSC1L1*; *NDRG1* (N-myc downstream regulated 1) which is frequently altered in human cancers including HNSCC but whose roles remain controversial [50]; cell cycle genes *CCNE1*, *CDK6* and *E2F3*; and others listed in S1E Table. Notably, the most enriched functions among the amplified genes are protein kinase activity, with 15 serine/threonine kinases and 13 tyrosine protein kinases, and protease activity, with 10 serine proteases (S1E and S1G Table). This is consistent with TCGA’s human HNSCC study [1]. Examples of deleted genes in the canine tumors (Fig 2A) include cell cycle gene *CDKN2A*, one of the best known gene deletions in human HNSCC [1,2,6]; and protein phosphatase *PTPRD* and cadherin superfamily membrane *FAT1*, tumor suppressors [51,52] that are also frequently deleted in human HNSCC [1]. Furthermore, the most significantly enriched functions among the deleted genes are closely related to epithelial cell polarity including adhesion (18 genes), small GTPase regulator activity (19 genes), and cell junctions (11 genes) (S1F and S1G Table). This agrees with human HNSCC findings [1] and is consistent with the concept that loss of cell polarity is a hallmark of epithelial cancers such as HNSCC [53]. These observations support the dog-human molecular homology.

### RNA-seq analysis reveals dog-human homology in transcriptomic alterations

**Fig 3:**
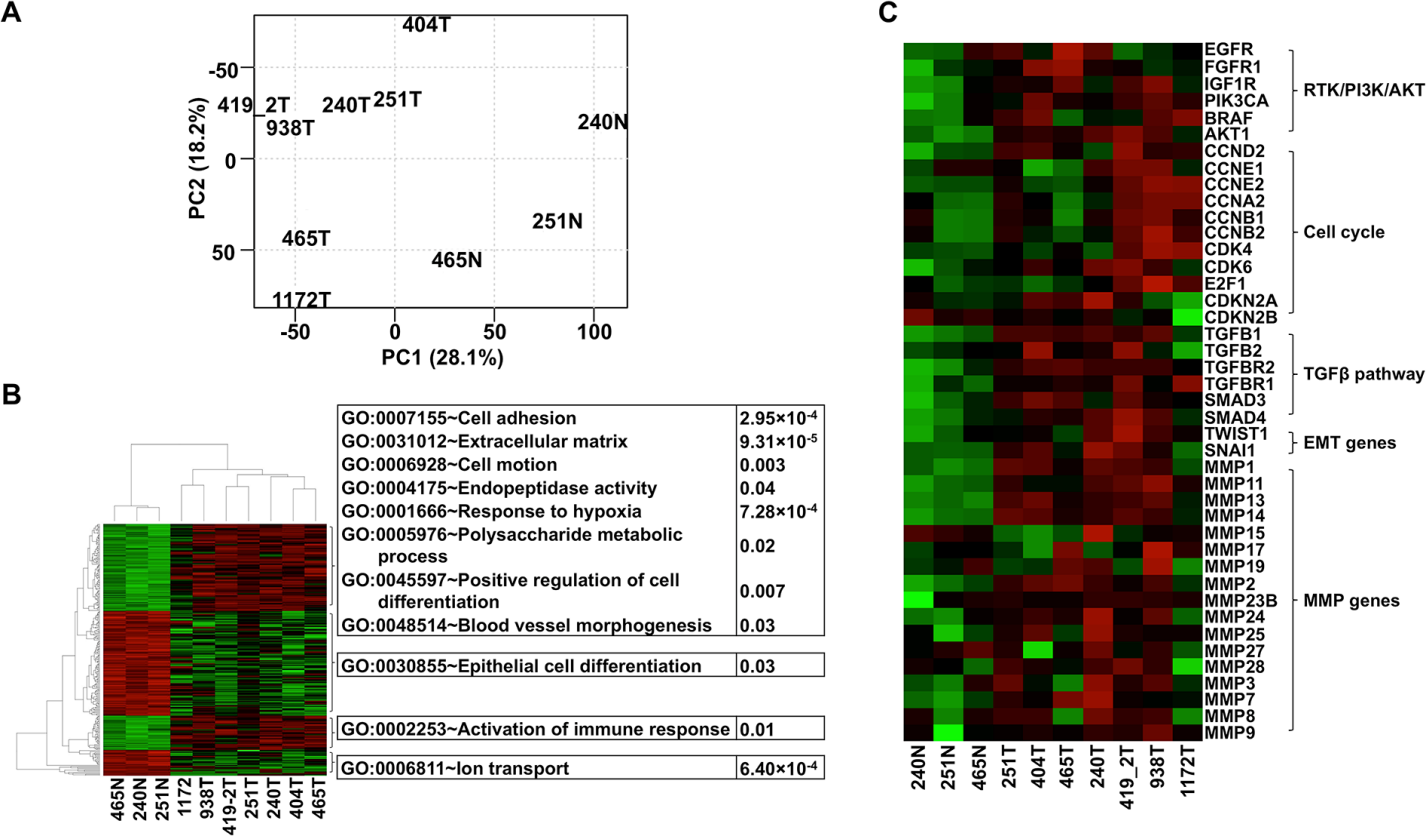
The same pathways are altered in canine oSCCs as in their human counterparts as revealed by RNA-seq analysis. (A) PCA with FPKM values of 12,478 total genes, each having FPKM ≥ 1 in at least one of the tumor (with ID followed by T, e.g., 240T) or normal (with ID followed by “N”, e.g., 240N) samples. The plot shows that tumors are separated from normal samples and tumors 1172T, 404T, and 465T are distinct from the other tumors. (B) Heatmap with the log_2_(*FPKM*) values of genes (255 total) that are differentially expressed between the tumors and the normal samples found by both DESeq and t-tests at FDR ≤ 0.1. The right panel illustrates the significantly enriched functions of each gene cluster indicated. Red denotes upregulation and green denotes downregulation. (C) Heatmap of genes from pathways and groups that are reported to be altered in human oSCC [1,2]. The heatmap is generated as described for (B).

To better understand alterations at the transcriptomic level, we performed RNA-seq on seven of the canine oSCCs and three matching normal tissue samples (S2A Table). The study further reveals canine tumor heterogeneity and supports dog-human molecular homologies. First, principle component analysis (PCA) separates the tumors from the normal samples (Fig 3A). More importantly, tumors 1172, 465, and 404 are distant from the other tumors in the PCA (Fig 3A), the significance of which will be discussed in later sections. Second, compared to the normal samples, the genes upregulated in the tumors are enriched in functions including: 1) cell adhesion/motility, extracellular matrix, and endopeptidase activity; 2) hypoxia and polysaccharide metabolic processes; 3) blood vessel morphogenesis and cell differentiation; and 4) immune response (Fig 3B and S2C–S2G Table). As in human cancers, these functions facilitate canine tumor cell proliferation and invasion.

We followed published strategies [2,46] to identify over/under-expressed genes. Consistent with CNA findings (Fig 2A), more genes are overexpressed than underexpressed in all tumors except tumor 404 (S2B Table). Importantly, genes recurrently overexpressed among the tumors are significantly enriched in functions associated with cell cycle (e.g., cytoskeleton, spindle, centrosome, kinetochore, etc.), protein kinase activity (e.g., *PTK2*, *TEC*, *CHEK2*, etc.), nucleolus, and mRNA and ncRNA processing. Recurrently underexpressed genes are, however, significantly enriched in functions related to cell junctions (e.g., 9 tight junction genes), mitochondria (e.g., respiratory chain and oxidative phosphorylation), serine protease inhibitors, and apoptosis. These functions promote cancer cell proliferation and invasion, consistent with human cancer findings [1].

**Fig 4:**
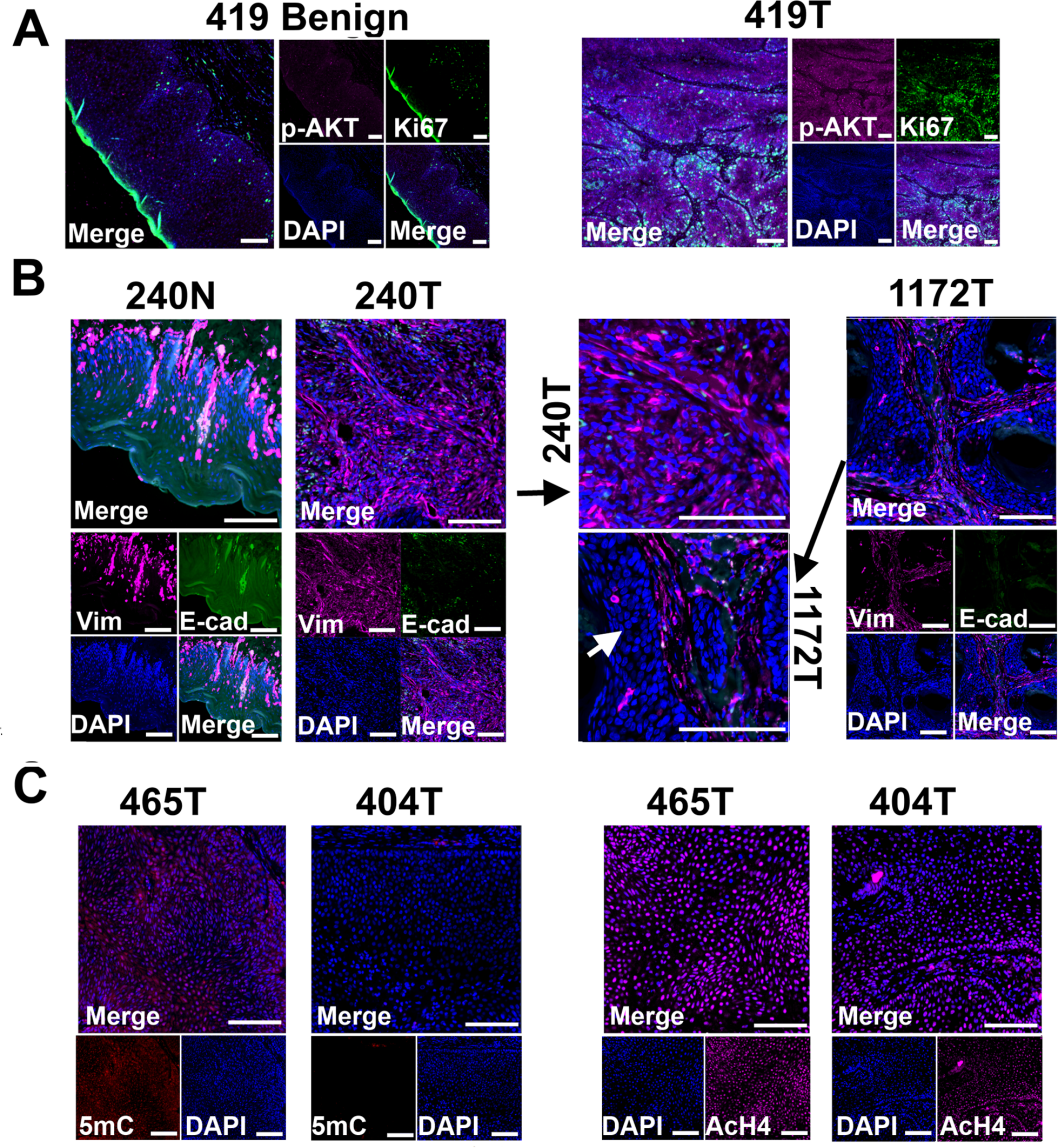
Tissue immunofluorescent staining supports conclusions from RNA-seq studies shown in Fig 3. (A) Phospho-AKT (p-AKT) is more strongly expressed in tumor cells than in benign cells of canine case 419, indicating the activation of PI3K/AKT pathway in the tumor cells. Tumor 419 has highest *AKT1* mRNA expression level based on RNA-seq analysis (Fig 3C). (B) In case 240, tumors cells (240T) express higher level of mesenchymal marker vimentin (Vim) but lower level of epithelial marker E-cadherin (E-cad) when compared to normal squamous cells (240N). Many proliferating tumor cells (e.g., those pointed by white arrow) of case 1172 however do not express vimentin. These are consistent with RNA-seq analyses (Fig 3C). (C) Tumor 404 has significantly decreased 5-methylcytosine (5mC) staining and somewhat decreased acetyl-H4 staining (AcH4), compared to tumor 465. This is consistent with the PCA result shown in Fig 3A indicating them being two distinct tumors. Representative confocal images are shown; scale bar, 50 μm.

Critically, we found the same genes and pathways altered in these canine cancers as reported in human HNSCCs [1,2]. For example, gene members of the receptor tyrosine kinase (RTK)/ PI3K/AKT pathway such as *EGFR*, *PIK3CA*, *BRAF*, and *AKT1* are recurrently overexpressed among the canine tumors (Fig 3C). *AKT1* is especially noteworthy because its expression level in each tumor is consistently higher than in each normal sample by 2-4-fold (Fig 3C). The PI3K/AKT pathway is indeed activated in the tumor cells as revealed by immunostaining with phospho-AKT (Fig 4A). Cell cycle is also altered, as evidenced by overexpression of multiple cyclin genes, *CDK4*, *CDK6* and *E2F1*; as well as underexpression of *CDKN2A* and *CDKN2B* (Fig 3C). These pathway alterations could promote cancer cell proliferation.

Another pathway affected is TGFβ signaling, as evidenced by the recurrent overexpression of *TGFB1*, *TGFB2*, *TGFBR2*, *TGFBR1*, *SMAD3* and *SMAD4* in the canine tumors (Fig 3C). Other notable changes include at least 12 matrix metalloproteinase (MMP) genes, whose expression increased by hundreds to thousands fold in at least one tumor (Fig 3C). Likewise, epithelial to mesenchymal transition (EMT) genes *TWIST1* and *SNAI1* are also recurrently overexpressed (Fig 3C). These observations indicate EMT in the canine tumors, which is confirmed by immunofluorescent staining showing increased expression of the mesenchymal marker vimentin and decreased expression of the epithelial marker E-cadherin in the tumor cells, compared to the normal squamous cells (Fig 4B). Activation of these genes and pathways would facilitate the invasion of tumor cells into adjacent tissues. Interestingly, tumor 1172 appears to be an exception to the above observations (Figs 3C and 4B), which will be revisited in the DISCUSSION section.

Finally, two canine oSCCs appear to be canine papillomavirus (CPV)-positive, based on the detection of papillomavirus sequences, specifically CPV7 E2/E4 sequences in tumor 1172 and sequences with high homology (>90%) to human papillomavirus HPV77 E2/E4 in tumor 465, among their RNA-seq reads (S2H Table). However, unlike TCGA’s HPV-positive HNSCCs that harbor thousands of viral RNA-seq reads per sample [1], we only identified a few viral RNA-seq reads for the two canine tumors (S2H Table). This indicates that the infection is likely latent, or the CPV genome(s) infected has/have not been sequenced (we were able to download 170 HPV genomes but only 15 CPV genomes from the PaVE database at pave.niaid.nih.gov at the time of the analysis).

### RNA-seq analysis reveals dog-human homologies in sequence mutation

**Fig 5:**
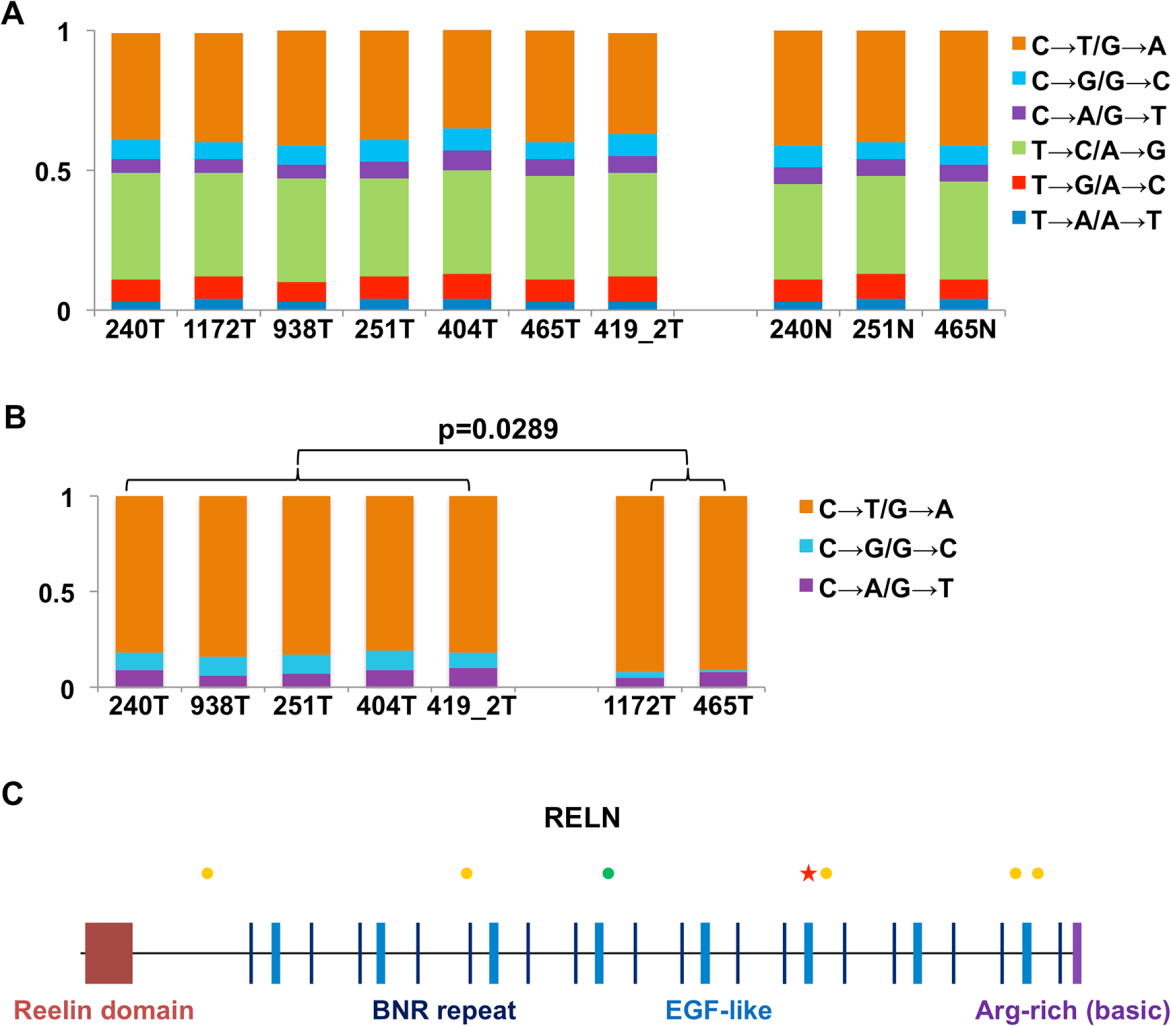
RNA-seq analysis reveals dog-human homology in sequence mutations. (A) The fractions (the Y-axis) of base substitution types in the canine samples (the X-axis) detected by RNA-seq when compared to the dog reference genome [33]. (B) Base transversions are significantly more prevalent in CPV-negative tumors than CPV-positive tumors at CpG sites. (C) Synonymous (green dots) and non-synonymous (yellow dots) substitutions, and a nonsense mutation (red star) uncovered in the *RELN* gene in tumor 404.

We took advantage of RNA-seq data to examine sequence mutations in the canine samples. Briefly, to achieve more accurate mutation-finding, we utilized only coding regions with 30-300X RNA-seq read coverage, which distribute across the genome and amount to 4–6 Mb sequences in total per sample (S2I Table). The analysis again reveals dog-human homologies. First, base transitions C↔T/G↔A dominate base transversions in all samples (Fig 5A and S2I Table), indicating similar mutation mechanisms in both species. At CpG sites, the two CPV-positive canine tumors harbor significantly more transversions compared to the CPV-negative tumors (Fig 5B and S2I Table), consistent with human findings [1]. However, we did not observe a predominance of mutations at TpC sites in CPV-positive canine tumors (S2I Table), unlike the human study [1]. Second, the analysis uncovered a somatic mutation, E233K, in *TP53*. Similarly, consistent with human studies [3,4], genes *FAT1*, *FAT2*, *UBR2*, *TNC* and others were found to be mutated in the canine tumors (S2I Table).

As an example, Fig 5C shows the mutations found in the gene *RELN* in canine tumor 404, including one nonsense, five non-synonymous and one synonymous changes. Importantly, *RELN* is significantly mutated in human HNSCC [1,4], non-small cell lung cancer of smokers [54], and acute lymphoblastic leukemia [55]. *RELN* encodes an extracellular matrix glycoprotein which is known to control cell–cell interactions to regulate neuronal migration and positioning in the developing brain. Thus, alteration of *RELN* may contribute to tumor cell invasion and spread in both the human and the dog.

### Driver-passenger discrimination via dog-human comparison for human 8q

**Fig 6:**
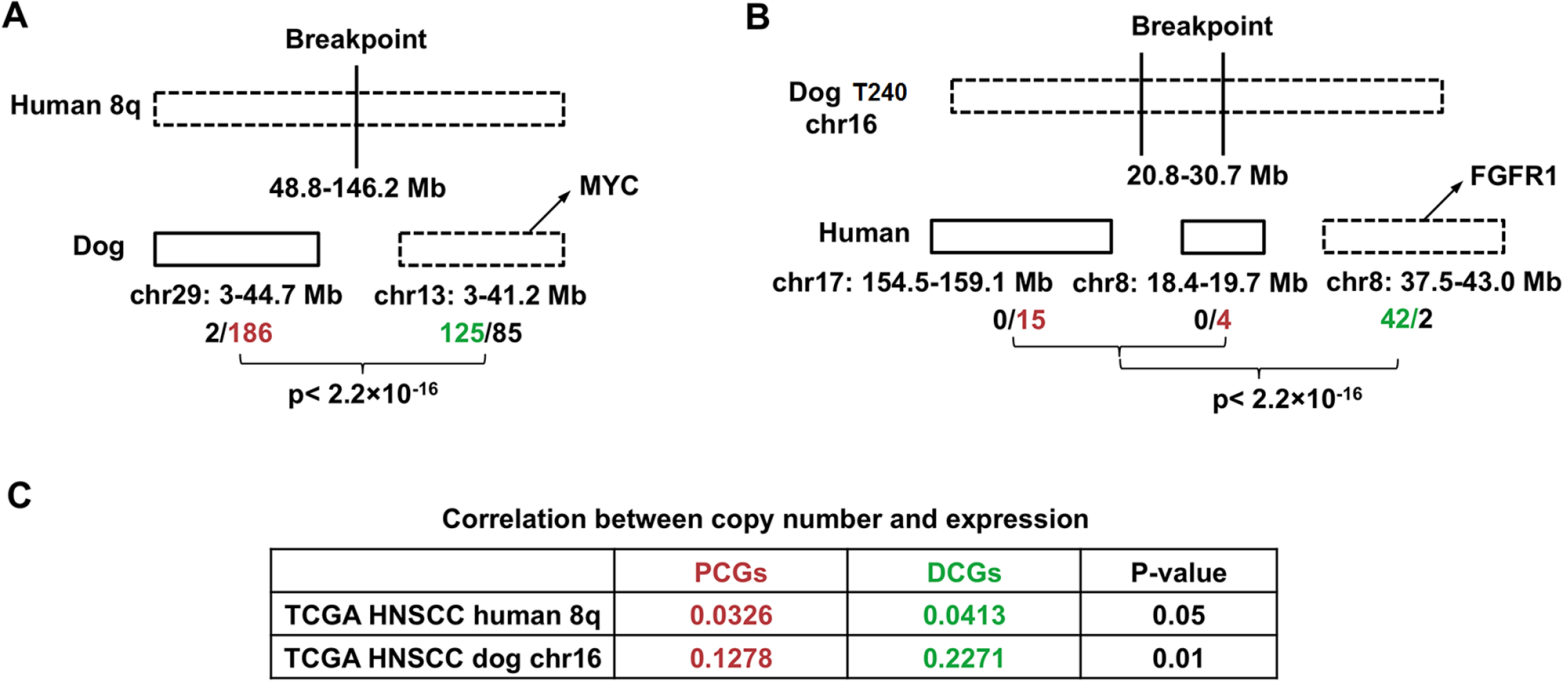
Pilot HNSCC driver–passenger discrimination via human–dog comparison. (A) Driver-passenger discrimination of human 8q. Human 8q is broken into dog chromosomes 29 (chr29) and 13 (chr13), with the numbers indicating the sequence coordinates. In human HNSCCs, the entire human 8q and all genes encoded (398 total) are recurrently amplified, as represented by a broken lined bar. In canine tumors, however, only chr13 is significantly amplified resulting in 125 genes being amplified and 85 genes unchanged, compared to only 2 genes amplified and 186 genes unchanged for chr29. The 125 amplified genes on chr13, including *MYC*, are considered as driver candidate genes (DCGs; in green), whereas the 186 unchanged genes on chr29 are deemed passenger candidate genes (PCGs; in red). The p-value shown is obtained with Fisher’s exact test; see S1H Table. (B) The 10Mb amplicon on chromosome 16 in tumor 240 (Fig 2B) is broken into three distinct regions in the human genome as shown. Only one region is significantly amplified in human HNSCC according to TCGA [1], and the amplified genes encoded there, including known driver *FGFR1* [1], are considered as driver candidate based on our dog-human comparison strategy [30]. The p-value shown is obtained with Fisher’s exact test, see S1H Table. (C) The correlation between copy number status, represented by a gene’s $\log_{2}\frac{Tumor}{Normal}$ value, and mRNA expression level, represented by a gene’s log_2_(*FPKM*) from RNA-seq or log_2_(*intensity*) from SNP arrays. The data are from TCGA [1]. The p-values shown are obtained with Hotelling's t-squared tests, see S1H Table.

The dog-human molecular homologies described above rationalize the use of the dog-human comparison strategy for driver-passenger discrimination as described [30] for HNSCC. Because of the small sample size of canine tumors, we tested this strategy only on human 8q, one of the most recurrently amplified regions in human HNSCC [1,2] and other cancer types. Due to interspecies genomic rearrangements, human 8q is broken into two dog chromosomal regions, which include chromosome 29 and the first 38Mb of chromosome 13 (Fig 6A). Notably, the entire human 8q is significantly amplified among TCGA’s 449 human HNSCCs (FDR < 10^–6^), leading to the amplification of all of its 398 genes (Figs 6A and S1). In canine tumors, however, only the chromosome 13 region is significantly altered, resulting in the amplification of 125 genes out of 210 total at FDR < 0.2. For chromosome 29 in contrast, merely 2 genes out of 188 total are amplified. These numbers significantly differ (p < 2.2×10^–16^) (Fig 6A). Thus, based on our strategy [30], amplified genes (125 total) on chromosome 13 are considered as driver candidate genes (DCGs), while unchanged genes on chromosome 29 (186 total) are deemed passenger candidate genes (PCGs) (Fig 6A and S1H Table).

We then performed several analyses to examine the differences between the DCGs and PCGs identified. First, a significantly better correlation between the copy number status and the mRNA expression level was observed for DCGs than PCGs, using data from TCGA [1] (Fig 6C and S1H Table). This indicates that amplification of DCGs is more functionally relevant than amplification of PCGs. Second, significantly more DCGs are mutated than PCGs, based on published human HNSCC studies [3] (p < 0.0081) (S1H Table). Lastly, well known cancer driver genes such as *MYC* are among the DCGs.

Conversely, we also tried the dog-human comparison strategy on the10Mb amplicon of canine tumor 240 shown in Fig 2B. In the human genome, the 10Mb region is broken into three pieces, including human chromosome 7: 154.5–159.1Mb; chromosome 8: 18.3–19.7Mb and 37.5–43.1Mb. Based on TCGA data [1], while 42 out of the 44 genes of chromosome 8: 37.5–43.1Mb are significantly amplified, none of the genes of the remaining two human regions is amplified or deleted (Fig 6B). Thus, based on our strategy [30], the chromosome 8: 37.5–43.1Mb region is more likely to harbor cancer drivers, which indeed encodes known driver gene *FGFR1* [1].

## Discussion

In this pilot study, we performed the first genome-wide characterization of spontaneous canine HNSCC. Although the sample size (12 cases including 9 oral) is small, the study reveals a complex alteration profile for canine HNSCC and a strong dog-human molecular homology. Our results indicate that spontaneous canine HNSCCs more accurately represent their human counterparts in various aspects than the current widely-used models. These canine cancers better model human HNSCCs in cancer cell origin and tumor microenvironment (compared to cell line and xenograft models), molecular heterogeneity and complexity (compared to genetically engineered mouse models), and histopathology and cancer biology (compared to carcinogen-induced models). If our conclusions are validated with a large sample size study, spontaneous HNSCCs in pet dogs could contribute significantly in basic and translational cancer research.

### Spontaneous canine HNSCCs recapitulate many molecular features of their human counterparts

As described throughout the Results section, our genomic and transcriptomic studies support strong dog-human molecular homologies for HNSCC at various levels. For large genomic changes, the two species share a similar CNA landscape, with some tumors harboring extensive CNAs while others being nearly CNA-free [1,2] and with large amplicons that likely harbor cancer drivers discovered. Furthermore, unlike canine mammary cancers [21], no potential oncogenic chimeric fusion genes are found in these canine oSCCs, consistent with very few such genes being reported for human HNSCC [1]. At the individual gene level, numerous amplified/deleted, over/under-expressed, or mutated genes are shared between the two species (e.g., *FADD* amplification, *CDKN2A* deletion, *FAT1* mutation, etc.). The altered genes are also enriched in the same functional groups, including protein kinase or protease activity for amplified/overexpressed genes, as well as cell adhesion and other epithelial polarity related functions for deleted/underexpressed genes. At the pathway level, both species show alterations in cell cycle, RTK/PI3K/AKT signaling, and TGFβ signaling and EMT.

Despite the strong homology described above, a few differences are also observed. For example, *TP53* mutation is more frequent in human HNSCC [1] than in these canine tumors, which is also true for *NOTCH1*. This difference however could be due to our small sample size and/or using RNA-seq instead whole exome-sequencing for mutation discovery. Another discrepancy is that more canine HNSCCs appear to be CNA-free when compared to their human counterparts, which again needs to be validated with a large sample size study.

Our study indicates that canine tumors 1172 and 465 are CPV-positive but the infection is likely latent (or the CPV genome(s) infected has/have not been sequenced). Both tumors (especially tumor 1172) are distinct from other canine oSCCs in gene expression. Importantly, consistent with the finding that HPVs infect the basal layer of squamous epithelium [56], tumor 1172 exhibits features indicating basal stem cell origin. Unlike other canine oSCCs, tumor 1172 overexpresses the pluripotent marker *SOX2* and at least 20 homeobox genes that are associated with embryonic morphogenesis, but does not express EMT markers in many of its tumor cells. These observations again reveal extensive heterogeneity of canine HNSCCs and a strong homology to their human counterparts.

The dominance of C↔T changes over other base substitution types indicates that deamination of C to U/T is a major sequence mutation mechanism in dogs as in humans. This result is consistent with aging being a risk factor for HNSCC development in both species [57]. Furthermore, analogous to the human [1], base transversions at CpG sites are more dominant in CVP-negative tumors than in CPV-positive tumors. However, unlike the human [1], CPV-positive tumors do not show predominance of mutations at TpC sites, which needs further study to resolve.

**Fig 7:**
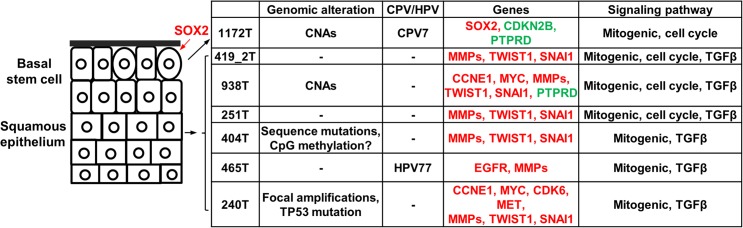
The proposed carcinogenic mechanisms of canine oSCCs investigated. The cells of origin in these tumors are hypothesized as indicated, with oval cells representing the basal stem cells. Alterations at various levels are shown. For individual gene alterations, red represents gene amplification or overexpression, while green represents gene deletion or underexpression.

We hypothesize the carcinogenic mechanisms of the canine tumors based on our findings (Fig 7). Regarding cells of origin, unlike tumor 1172 discussed previously, we propose that other tumors arise from more differentiated cells of the squamous epithelium (Fig 7), because of overexpression of protease genes and EMT genes. At the genome level, we hypothesize that primary drivers include focal amplifications for tumor 240. Tumor 404 is also noteworthy, as it lacks genomic CNAs but has the largest number of underexpressed genes and appears to have a different epigenomic landscape, as shown by drastically reduced staining by 5-methylcytosine antibody (Fig 4C). While emphasizing that the significance and reasons of the observed DNA hypomethylation need further investigation, we hypothesize epigenomic aberrations as the drivers of tumor 404 (Fig 7). Finally, notable gene alterations and recurrently altered pathways are listed as cancer drivers. Fig 7 summarizes once again the complexity and heterogeneity of these canine cancers and their numerous homologies to human HNSCCs.

### If validated with a large sample study, spontaneous canine HNSCCs could be used for efficient cancer driver-passenger discrimination

The dog-human molecular homologies described above indicate that canine HNSCC share similar pathogenic pathways as their human counterparts, which, if validated with a large sample study, justifies the use of our established dog-human comparison strategy [30] for driver-passenger [58] discrimination for HNSCC. Indeed, our pilot human 8q study has shown that this approach is valid. The use of canine cancers in this regard is highly significant. For example, our analysis with the copy number data of TCGA’s 449 human HNSCC cases has found over 7000 amplified/deleted genes at FDR ≤ 0.05, including those harbored by both focal amplifications/deletions and broad (chromosomal arm level) gains/losses. Among these genes, some are drivers and some are passengers. Based on our analyses, a sample size of 449 tumors is already saturating, and studying additional human tumors no longer helps in determining which amplified/deleted genes are drivers and which are passengers. However, investigating about 90 canine tumors will reduce the number of driver candidates by at least half, according to our estimation, significantly reducing the workload of downstream functional validation which is time-consuming and expensive.

### If validated with a large sample size, spontaneous canine HNSCCs may bridge the gap between current preclinical models and human clinical trials

As discussed in the Induction section, a major obstacle in translational research is the lack of effective predictive models [9]. Current preclinical models, including cell culture and xenograft or genetically-induced rodent models, typically fail to represent the vast heterogeneity and complexity of human cancers and often do not predict clinical results. Thus, a cancer model that can bridge the gap between these preclinical models and human clinical trials is urgently needed.

Spontaneous canine HNSCCs could serve as such a much-needed translational model, if the dog-human molecular homologies concluded here are validated with a large sample study. Being molecularly complex and heterogeneous and recapitulating many molecular features of human HNSCC, these canine HNSCCs are more accurate representatives of their human counterparts than current cell line or rodent models. With an HNSCC incidence rate comparable to that in the human and a large pet dog population (>70 million in the US), coupled with less stringent FDA regulations governing pet clinical trials and increasingly improved resource and infrastructure in pet cancer research [18], spontaneous canine cancers have the potential to significantly accelerate the translation of basic research findings into successful clinical applications.

In summary, our study indicates that spontaneous canine HNSCCs better recapitulate the full spectra of the biology, histopathology, complexity, and heterogeneity of humans HNSCC than the current widely used models. However, we caution again that our sample size is small, and the conclusions drawn from this pilot study need to be validated with a larger sample size.

## Materials and Methods

### Canine tissue samples

Fresh-frozen normal and tumor tissue samples and formalin-fixed paraffin-embedded (FFPE) tissue sections of spontaneous canine HNSCCs were obtained from the Animal Cancer Tissue Repository of Colorado State University. Samples were collected from client-owned dogs that developed the disease spontaneously, under the guidelines of the Institutional Animal Care and Use Committee and with owner informed consent. The breed, age, histopathologic descriptions, and other information are provided in S1A Table. These data, along with our own H&E staining of each sample received, were reviewed by Dr. Angela E. Ellis, a canine cancer pathologist.

### Tissue dissection, DNA and RNA extraction, and PCR analyses

Tissue cryosectioning, H&E staining, and cryomicrodissection were performed as described [19] to enrich for tumor cells in tumor samples and squamous epithelial cells in normal samples. Genomic DNA and RNA were then extracted from the dissected tissues using the AllPrep DNA/RNA Mini Kit (cat. no. 80204) from Qiagen. Only samples with a 260/280 ratio of ~1.8 (DNA) or ~2.0 (RNA) and showing neither degradation nor other contamination on the agarose gels were subjected to further analyses.

### aCGH analyses

Canine aCGH experiments were conducted at the Florida State University Microarray Facility, with 385K canine CGH array chips from Roche NimbleGen Systems, Inc. as previously described [19]. Briefly, each array chip contains about 385,000 probes of approximately 50 bp long oligos selected from unique sequences in the canFam2 genome, providing an average resolution of one probe every 5–6 kb across the canine genome. Tumor DNA purified from the dissected tumor tissue sample and paired normal DNA purified from matching normal tissue, skin, or blood of the same dog patient (S1A Table) ware hybridized to a chip, following the manufacturer’s instructions. This was performed for each canine case except for case 573 (a male), of which a normal sample from the same dog was unavailable and hence normal DNA from another dog (female) of the same breed was used instead (S1A Table). Because of tumor 573, chromosome X was excluded from the CNA-finding to minimize artifacts.

CNAs were identified as previously described [19]. Briefly, CNAs in each tumor were detected by analyzing the log_2_-ratios using a software program called SEG, which we developed to more effectively decipher high density oligo aCGH data for CNA finding. As described in our previous publication [19], SEG first identified change-points at the chromosomal level via dynamic programming and then detected CNAs at the whole genome level using false discovery rate (FDR)-controlled procedure [59]. For this study, we set the desired FDR to 0.01, the total probe number cutoff to 3, and the log_2_-ratio mean cutoff to 0.4 for CNA discovery.

Significantly amplified/deleted genes were identified by GISTIC [60] as previously described [19,21]. For the human, level 3 data of SNP array analyses of 449 human HNSCC cases were downloaded from TCGA site (cancergenome.nih.gov/), and GISTIC [60] were used to detect amplified/deleted genes, in combination with published findings [1].

### Paired-end RNA-seq

Sequencing was conducted using the Illumina platform, following the protocols from the manufacturer. RNA-seq was performed at the HudsonAlpha Institute for Biotechnology or the BGI-America, yielding 48 to 66 million paired-end sequence reads of 50bp or 49bp per sample (S2A Table).

### RNA-seq data analysis

RNA-seq data analyses were performed as described [21]. Briefly, read pairs were aligned to the dog reference genome [33] canFam2 with TopHat v2.0.5 (tophat.cbcb.umd.edu). The uniquely mapped pairs were used to quantify a gene’s expression level by calculating its FPKM (fragments per kilobase of exon per million mapped fragments) value, using Cufflinks (cufflinks.cbcb.umd.edu) with default parameters and the canine gene annotation downloaded from the University of Santa Cruz (UCSC) genome site. Base substitutions were identified with VarScan2 (varscan.sourceforge.net) in coding regions with RNA-seq read coverage ranging from 30X to 300X. Over/underexpressed genes in cancers were identified as described [2,46], i.e., genes whose expression levels are outside 95% confidence internals of the expression of samples that are diploid in that gene. Differentially expressed genes between two groups of samples were identified by DESeq [61] and t-tests. Gene functional annotation and enrichment analyses were achieved using the DAVID Functional Annotation tool at david.abcc.ncifcrf.gov.

Viral RNA-seq reads were identified using a similar approach as VirusSeq (odin.mdacc.tmc.edu/~xsu1/VirusSeq.html). After aligning RNA-seq reads to the reference genome (canFam2) with TopHat v2.0.5, the unmapped reads of each sample were realigned to the HPV and CPV genomes downloaded from the PaVE database (pave.niaid.nih.gov/) with Bowtie2 v2.2.3 and BWA v0.7.10.

### Immunofluorescence

Immunofluorescent staining was performed with 5-μm FFPE tissue sections as described [21,62]. Primary antibodies used include those against E-cadherin (R&D Systems; AF648), vimentin (Abcam; ab92547), phospo-AKT (Ser473) (Cell Signaling; 4060), acetyl-H4 (Millipore; 06–866), 5-methylcytosine clone 33D3 (Millipore; MABE146), and Ki67 (Life Technologies; 08–0156). Alexa Fluor488–, 647—or 594–conjugated secondary antibodies are from Jackson ImmunoResearch. Images were taken with a Zeiss LSM 710 confocal microscope.

### Accession numbers

RNA-seq data have been submitted to the NCBI SRA database under accession number SRP046723. aCGH data have been submitted to the GEO database under accession number GSE61231.

## Supporting Information

S1 Table Case information and aCGH analysis results. A. Canine HNSCC case information. B. CNAs of canine HNSCCs determined by aCGH analysis, and the significantly enriched functions and pathways of amplified/deleted genes found by DAVID (p≤0.05). C. Genes harbored by the focal amplification on the chromosome 16 (chr16:20874453–30664857) in tumor 240. D. Significantly enriched functional groups of amplified and overexpressed genes in tumor 240 by DAVID (p≤0.05). E. Amplified genes in canine HNSCCs found by aCGH analysis. F. Deleted genes in canine HNSCCs found by aCGH analysis. G. Recurrently amplified/deleted genes identified by GISTIC in canine HNSCCs at FDR< = 0.2. H. Evaluation of the difference between DCGs and PCGs identified on human 8q.
(XLS)

S2 Table RNA-seq analysis results. A. Canine oSCC RNA-seq read mapping summary. B. Low, normal, and high gene expression groups with a 95% confidence interval cutoff following a published method. C. Differentially expressed genes between tumors and normal samples found by both DESeq and t-test at FDR<0.1. D-G: Functional Annotation of the 1^st^ (D), 2^nd^ (E), 3^rd^ (F), and 4^th^ (G) group of genes differentially expressed between tumors and normal samples in Fig 3B by DAVID (p≤0.05). H. Canine oSCC RNA-seq reads that match sequences from the CPV or HPV genomes by Tophat. I. Sequence mutations found in canine samples using RNA-seq reads.
(XLS)

S1 Fig Recurrent CNAs found in TCGA’s 449 HNSCC samples by GISTIC [60]. The images were drawn as described [19], with each line representing a human chromosome and vertical lines above/below the chromosome indicating amplifications (red) or deletions (blue) respectively. Black boxes represent the telomeres and centromeres in human chromosomes.
(TIF)

S2 Fig Base pair size distributions of amplifications and deletions found in canine HNSCCs. (TIF)
