# Supplementary figures and images for: Canine Spontaneous Head and Neck Squamous Cell Carcinomas Represent Their Human Counterparts at the Molecular Level
Source: PLoS Genet. 2015 Jun 1;11(6):e1005277. doi: 10.1371/journal.pgen.1005277 (PMC4452692; doi:10.1371/journal.pgen.1005277)

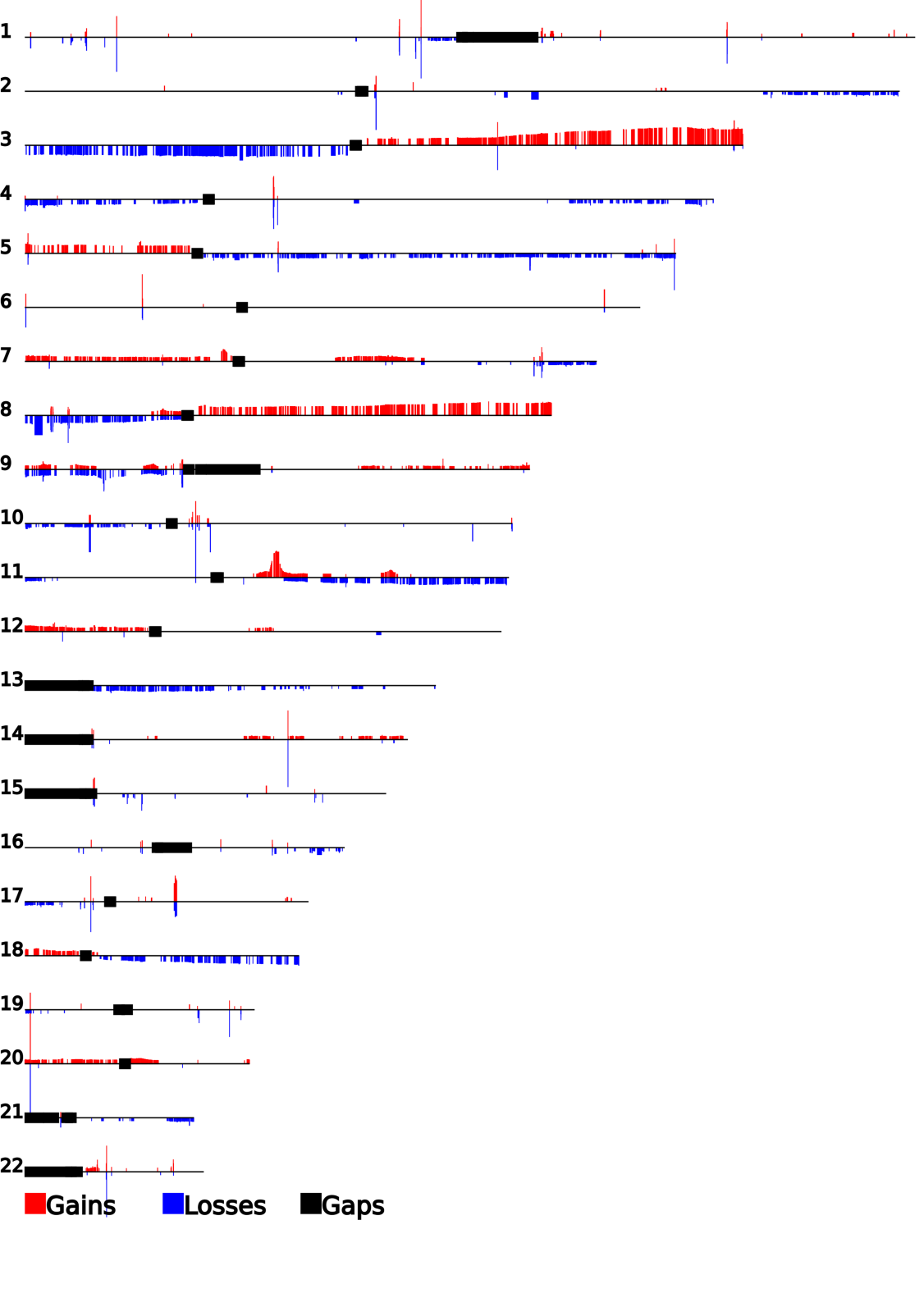

Supplement: S1 Fig — The images were drawn as described [19], with each line representing a human chromosome and vertical lines above/below the chromosome indicating amplifications (red) or deletions (blue) respectively. Black boxes represent the telomeres and centromeres in human chromosomes. (TIF) [file pgen.1005277.s003.tif]

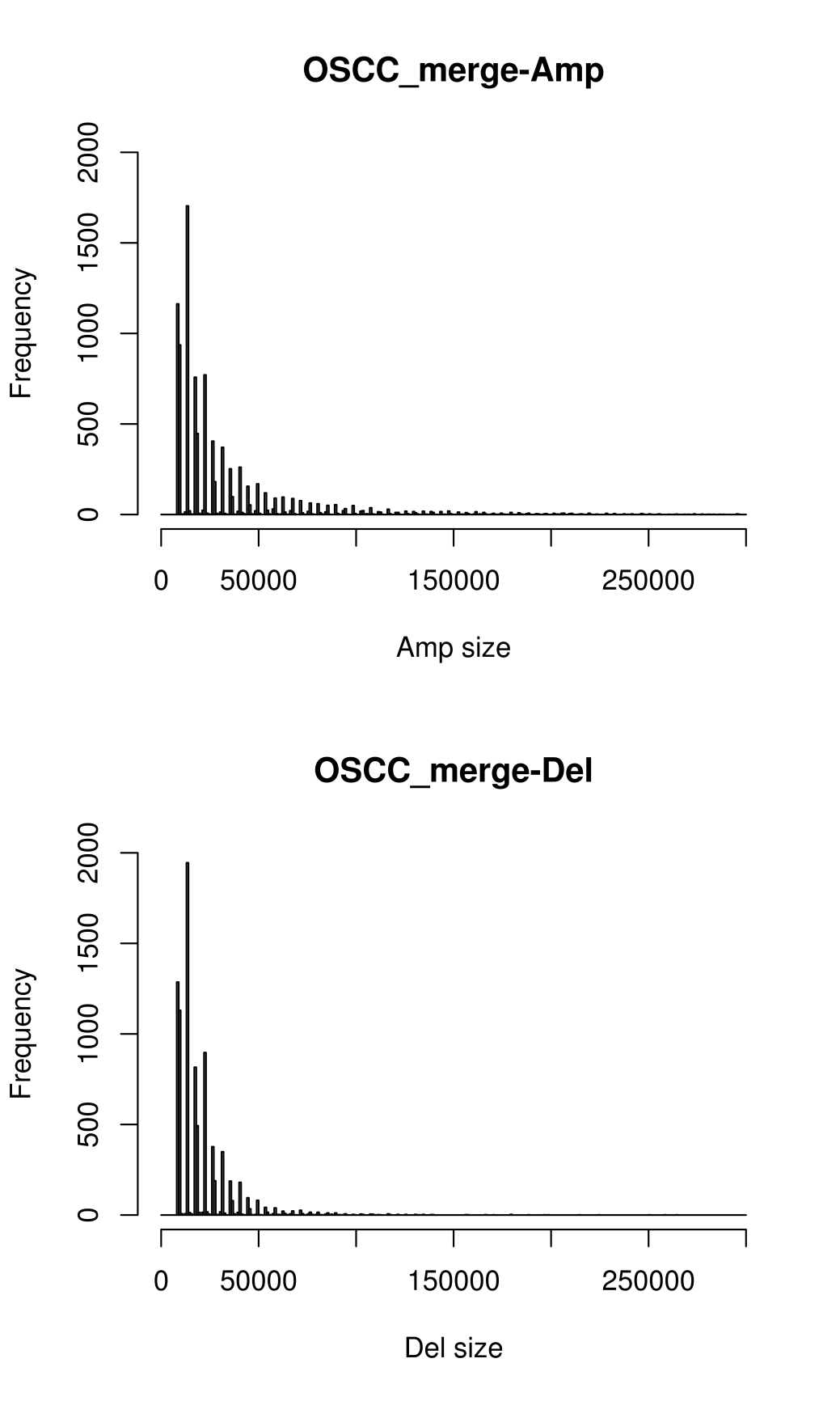

Supplement: S2 Fig — (TIF) [file pgen.1005277.s004.tif]
